# HIV testing among pregnant women living with HIV in India: are private healthcare providers routinely violating women’s human rights?

**DOI:** 10.1186/1472-698X-14-7

**Published:** 2014-03-24

**Authors:** Purnima Madhivanan, Karl Krupp, Vinay Kulkarni, Sanjeevani Kulkarni, Neha Vaidya, Reshma Shaheen, Sean Philpott, Celia Fisher

**Affiliations:** 1 Department of Epidemiology, Robert Stempel College of Public Health and Social Work, Florida International University, 11200 SW 8 Street, HLS 390W2, Miami, FL 33199, USA; 2 Public Health Research Institute of India, 89/B, 2nd Cross, 2nd Main, Yadavgiri, Mysore 560020, India; 3 Health Promotion and Disease Prevention, Robert Stempel College of Public Health and Social Work, Florida International University, Miami, USA; 4 Prayas, Amrita Clinic, Athawale Corner, Karve Road, Deccan Gymkhana, Pune 411 004, India; 5 The Bioethics Program, Union Graduate College, New York, USA; 6 Center for Ethics Education, Fordham University, Bronx, New York, USA

**Keywords:** India, HIV testing, Antenatal care, Confidentiality, Diagnosis, Qualitative research, Perinatal transmission

## Abstract

**Background:**

In India, approximately 49,000 women living with HIV become pregnant and deliver each year. While the government of India has made progress increasing the availability of prevention of mother-to-child transmission of HIV (PMTCT) services, only about one quarter of pregnant women received an HIV test in 2010, and about one-in-five that were found positive for HIV received interventions to prevent vertical transmission of HIV.

**Methods:**

Between February 2012 to March 2013, 14 HIV-positive women who had recently delivered a baby were recruited from HIV positive women support groups, Government of India Integrated Counseling and Testing Centers, and nongovernmental organizations in Mysore and Pune, India. In-depth interviews were conducted to examine their general experiences with antenatal healthcare; specific experiences around HIV counseling and testing; and perceptions about their care and follow-up treatment. Data were analyzed thematically using the human rights framework for HIV testing adopted by the United Nations and India’s National AIDS Control Organization.

**Results:**

While all of the HIV-positive women in the study received HIV and PMTCT services at a government hospital or antiretroviral therapy center, almost all reported attending a private clinic or hospital at some point in their pregnancy. According to the participants, HIV testing often occurred without consent; there was little privacy; breaches of confidentiality were commonplace; and denial of medical treatment occurred routinely. Among women living with HIV in this study, violations of their human rights occurred more commonly in private rather than public healthcare settings.

**Conclusions:**

There is an urgent need for capacity building among private healthcare providers to improve standards of practice with regard to informed consent process, HIV testing, patient confidentiality, treatment, and referral of pregnant women living with HIV.

## Background

In India, 38% of the estimated 2.4 million people living with HIV are females [1]. Approximately 49,000 of these women become pregnant and deliver each year, many without antenatal care, antiretroviral prophylaxis to prevent HIV transmission to their infants, or institutional delivery [2,3]. While the government of India has made some progress increasing the availability and accessibility of prevention of mother-to-child transmission of HIV (PMTCT) services, only 23% of pregnant women received an HIV test in 2010, and about one-in-five HIV-positive pregnant women received anti-retroviral treatment to prevent transmission of the HIV virus to their infants [3-5].

The World Health Organization (WHO) has explicitly enumerated human rights protections required for HIV testing [6]. These basic rights entitle all people undergoing HIV testing to receive the 5Cs: Informed Consent, Confidentiality, Counseling, Correct Result, and Connection-to-care. These standards and the key principles they represent have been further enumerated by Joint United Nations Program on HIV/AIDS (UNAIDS) [7]:

1. Consent: People being tested for HIV must give informed consent prior to being tested. They must be informed of the process for HTC (HIV Testing and Counseling), the services that will be available depending on the results, and their right to refuse testing. Mandatory or compulsory testing is never appropriate, regardless of where that coercion comes from: health-care providers, partners, family members, employers, or others.

2. Confidentiality: Testing services must be confidential, meaning that the content of discussions between the person tested and the health-care worker, testing provider, or counselor, as well as the test results, will not be disclosed to anyone else without the consent of the person tested.

3. Counseling: Testing services must be accompanied by appropriate and high-quality pre-test information or pre-test counseling, and post-test counseling.

4. Correct Result: Testing must be performed and quality assurance measures followed according to internationally recognized testing strategies, norms, and standards based on the type of epidemic. Results must be communicated to the person tested unless that person refuses the results.

5. Connection-to-Care: Connections to HIV prevention, treatment & care and support services should be supported through concrete and well-resourced patient referral, support, and/or tracking systems.

These standards also place the obligation on the healthcare provider to deliver testing in a non-discriminatory manner regardless of a patient’s HIV status, risk behavior, gender or sexual orientation. They also state that testing shall not be coercive or result in a denial of or diminution of services [8].

As an adopter of the Declaration of Commitment on HIV/AIDS in the United Nations General Assembly Special Session (UNGASS) on HIV/AIDS in June 2001, India is obligated to protect the basic rights of people undergoing HIV testing [9]. The Government of India and the National AIDS Control Organization have reaffirmed these basic rights in their HIV Testing and Operational Guidelines for Integrated Counseling and Testing Centers [10,11]. Beyond these fundamental obligations, Bar *et al.* have suggested that there are other important reasons for protecting human rights in HIV testing [12]. Without a safe testing environment where women can learn their status without fear of stigma, discrimination and violation of their rights, the Government of India risks both the effectiveness and sustainability of their HIV prevention programs. Unless benefits outweigh the potential harms, women will go to great lengths to avoid HIV testing, defeating both the purpose and usefulness of PMTCT programs [13].

The objective of the study was to better understand the experiences and perceptions about the HIV testing process during the antenatal care. This paper describes the findings from a qualitative study carried out among HIV-positive women who recently attended and delivered in public hospitals in Mysore and Pune, India.

## Methods

### Study sites

The study was carried out in Mysore (Karnataka) and Pune (Maharashtra) districts in India. These states are both considered high HIV prevalence states by the Government of India National AIDS Control Organization (NACO) and account for about 28% (670,000) of the total people living with HIV/AIDS (PLWHA) in India [14]. As of 2011 census, the total population of Mysore and Pune Districts were 2,994,744 and 9,426,959 respectively [15]. Mysore District has an urban population of 41% and a literacy rate of 87%. Pune District is 58% urban and has a literacy rate of 81%. According to the 2011 Census 76.8% of the population is Hindu, 19% Muslim and 2.8% belong to other religions in Mysore. The population is 71% Hindu, 12% Muslim, and 17% other religions in Pune. *Kannada* and *Marathi* are the regional languages spoken in Mysore and Pune respectively.

### Community preparedness activities

Prior to commencement of the study, the study investigators presented and sought input from a Mysore based Community Advisory Board (CAB). The purpose of the study was to explore women’s perceptions about HIV testing and provision of PMTCT interventions. The investigators explained the research objective; methods proposed and overall conduct of the study. In addition, 2 community meetings were held to explain the research to the community members. During the community meetings the study staff made a brief presentation on the purpose and objectives of the study; answered questions and sought input and suggestions from the audience, which were then incorporated into the study wherever necessary. One of the main changes proposed by the CAB members was to not conduct focus group discussion of HIV positive women due to concerns of confidentiality. We changed the study methods from focus group discussions to in-depth interviews for HIV positive women.

### Study design

The research was conducted using a qualitative study design. Qualitative methods were chosen in order to allow for an in-depth exploration of reported procedures for HIV testing and provision of PMTCT, and the context that shaped participant perceptions of those processes. Qualitative methods also allow for a flexible and open-ended exploration of the topic to ensure that the investigator will not impose his/her own ideas or preconceptions [16].

### Participant recruitment

Participants included women who delivered a baby in the last two years and used the public health facility for their antenatal care. Potential participants were identified from HIV positive women support groups, nongovernmental organizations serving women living with HIV, National Rural Health Mission, and physicians working with Public Health Research Institute of India or Prayas Health Group. In order to be eligible for the study, they had to have been pregnant in the prior two years, received antenatal care and HIV testing, found to be infected with HIV, have the ability to speak *Marathi* or *Kannada*, be over 18 years of age, have the ability to undergo informed consent process and be willing to have the interview audio-recorded.

We reached out to 37 HIV positive women, 24 women expressed interest in the study, of which eighteen were eligible and fourteen HIV-positive recently delivered women were recruited. We planned to continue recruiting women till we reached saturation in the themes. Women expressing interest in the study were asked to speak with a study recruiter who met potential participants in a private location and provided a detailed description of the study including the interview process, duration, and type of information sought. Women were informed about the possibility of discomfort in recounting their experiences, the potential for breach of confidentiality, and the steps that the study investigator would take to minimize that risk. In addition, they were told that taking part in the study was voluntary, and refusal to participate or answer any question would not result in any penalty or loss of healthcare to which they were otherwise entitled. Finally, potential participants were read an informed consent statement, which they could sign or if unable to do so for any reason, imprint with a thumbprint, to show their consent. All interviews were carried out in-person by female interviewers, and respondents received rupees 150 (USD $3) as compensation for their travel expense and time.

### Protection of human subjects

The study protocol was reviewed and approved by Institutional Review Boards at Florida International University (Protocol # 012712–04), Miami; Prayas Health Group in Pune and Public Health Research Institute of India in Mysore (Protocol # 2012-01-22-14), India. All study participants underwent an informed consent process prior to data collection.

### Data collection

In-depth interviews lasting between 60 to 90 minutes were carried out to ensure that participants could share complex descriptions of their experiences and perceptions about the topics under study. An interviewer guide was utilized during data collection. It covered demographic information; general experiences with antenatal healthcare; specific experiences around HIV testing and counseling; and perceptions about their experiences during and following HIV testing as part of their antenatal care. The guide was informed by a human rights framework described by the Joint United Nations Program on HIV/AIDS and the World Health Organization [8]. Interviews explored women’s experiences and feelings around access to healthcare; confidentiality & privacy; HIV pre and post-test counseling; non-discrimination; links to PMTCT services and HIV management. All interviews were conducted by trained female interviewers in *Marathi* or *Kannada* in a conversational style. We stopped the interviews after the fourteenth participant as we had already reached saturation in themes and were not getting any new information. The interviews were audio-recorded, transcribed verbatim, and translated into English by an independent translator to ensure accuracy using methods described by Brislin [17].

### Data analysis

ATLAS.ti (Version 6.1, Scientific Software Development) was used to assist with analysis of the qualitative data. Interviews were compared and contrasted to ensure that a full range of views were captured. Data were analyzed using a framework described by Lacy and Luff in which pre-defined topics were used for analysis but the analysis had enough flexibility to explore new themes if they arose [18]. The first three stages of the analysis were familiarization (reading the transcripts multiple times), development of a set of themes defined by the human rights framework described above [8], or arising naturally in the course of the interviews; and indexing and summarizing the themes and sub-themes.

## Results

### Characteristics of participants

The reported age of the women ranged from 19 to 29 years, with a mean age of 23. All participants had delivered a live child within the prior two years and were currently married and living with their husbands. Half of the participants had no schooling and the remainder had one to nine years of education. Of the 14 participants, eleven were housewives and three worked as day laborers. Eleven of the 14 participants resided in rural villages and the remainder in either Pune or Mysore City. Nine women reported their religion as Hindu and the remainder Muslim. Only one of the 14 participants had an HIV diagnosis prior to seeking antenatal care in their most recent pregnancy.

In the following sections, we present the major themes that emerged during interviews among the participants who had undergone HIV testing during their most recent pregnancy.

#### The right to informed consent

Most participants reported that healthcare providers made no effort to gain informed consent for any of the procedures that were part of their antenatal care including HIV testing as required by the Declaration of Commitment on HIV/AIDS [9]. A description of their interactions with providers underlined an inherent power differential that appeared to make no allowance for patient autonomy. This was particularly true of rural women who appeared to be more willing to tolerate substandard or even abusive care in the belief that “some care was better than none”. One woman described the one-sided interaction she experienced with her provider in this way:

“I went to [name omitted] Nursing Home in Bannur. When I went there they did not tell what or where, nothing they told. They just write, prick, write, prick, and send. That’s all they do… There we did not come to know anything, this or that… Nothing we came to know… It was only when we were sent to the government hospital that they said I have HIV.”

Informed consent rests on the premise that patients are actively involved in their own healthcare [19]. In the process described by most participants, they were passive observers who were told little or nothing about the risks or benefits of the procedures they undergo. A women attending a large urban private hospital described communication from providers that was almost completely prescriptive:

Participant: I came with my husband for a scan [ultrasonogram]. I was already in the fourth month of pregnancy. They ask my information and gave me a case paper. They said go to that office and get the tests. Sister [nurse] took the blood.

Interviewer: And what did they say to you when you went to the office?

Participant: They didn’t say. They only took the case paper and sister took the blood.

Interviewer: So did you get the results that day?

Participant: Yes. They told us to wait. They came after one hour with a report.

Interviewer: What was on the report? What did they tell you when they gave you the report?

Participant: She did not tell. I don’t know what she wrote. Everything was written in English and I don’t know what it was. Must be for HIV… The sister said we had to go to the Big Hospital [government hospital]. It was there I found out that I have something called HIV.

Interviewer: So you did not ask what was in the report?

Participant: No, they did not tell us and we did not ask.

Almost all the women who initially attended private healthcare facilities reported similar experiences that could broadly be described as poor or non-existent communication from healthcare workers describing the reasons for HIV testing, or the meaning of a positive test result.

#### The right to confidentiality

Many of the participants reported a lack of confidentiality during HIV testing as required by national and international standards [9,10]. The violation of this right took several forms. The first was a testing process that several of the participants perceived as opening them up to discrimination, stigmatization, and ostracism. One woman described how the extended wait and interactions with the healthcare personnel singled her out to other women attending the hospital:

Participant: There were a lot of people and we sat down with them. After that they started calling us one by one, they called out our names, and then we went to them and they asked where do I reside, my house number, and they wrote down everything.

Interviewer: What did they tell you about why you were there?

Participant: Nothing. They drew the blood…we did not know the reason. They said that it has to be done in the third month [of pregnancy]. Then they took it…the blood.

Interviewer: And what happened next?

Participant: After a while they called every name and all the women went except me. They got up and left…but I was not called. After a while someone came up to me and said your test is spoiled. We need to do another. They took more blood and I waited some more time. During that time, other women came in for their test and also left, but not me. And when they talked to me next, they said you have to go to local [name omitted] Hospital and get medicines and they handed me a report.

Interviewer: What did the report say?

Participant: I could not know what was in it [participant was illiterate].

Interviewer: And how did you feel?

Participant: I was frightened at that time. What will happen now? What is this test for? What will happen to my baby? What will they do next? And all the other women were watching and they knew that something will happen to me.

Participants described how they were told about their HIV diagnosis in the presence of their family, staff and even patients. One participant described how she was told that she was infected with HIV in a busy physician’s office:

Participant: We had been waiting a long time and the sister [nurse] finally said you will need to talk to the doctor.

Interviewer: This was after the blood was drawn for your HIV test?

Participant: Yes. It was a long time after that.

Interviewer: What happened in the doctor’s office?

Participant: He had many people coming…getting his sign [signature]. He said that my test had HIV and I would have to take medicines for it.

Interviewer: There were other people in the room when he said this?

Participant: Yes.

Interviewer: Did he explain about HIV or tell you anything about preventing your infant from getting the disease?

Participant: No. He wrote something and said that I would have to get some medicines at the Government Hospital.

Many of the participants described how relatives came to know about their HIV status. One participant for instance, related how an outside testing laboratory called her home and left a message with her sister-in-law:

“They said there is something wrong with her blood and she has to come and pick up the report right away. They said she has to go to [name omitted] hospital, and get checked up. This is how the family knew it was not right.”

Another woman who was living in an extended family explained how her in-laws found out her HIV status:

“I went to this Pallahalli doctor, got the test done. Then I knew that I have HIV. Then I thought: What if my mother-in-law gets to know this? She will scold me, criticize me, thinking I knew this and did not tell. Later the doctor called her and then she started to look at me as her enemy.”

Either intended or unintended breaches of confidentiality appeared to be quite common experiences among this sample of women. In most cases, these appeared to be related more to the lack of privacy available in many Indian healthcare settings. While healthcare workers appeared not to be deliberately subjecting participants to discrimination, many of the women felt that the process singled them out and subjected them to stigma. In the most disturbing violations of human rights, women reported that physicians and healthcare workers intentionally informed family members about their HIV status without consulting or informing them. The implications of this finding are sobering since in India many women testing positive for HIV have been evicted from their homes, abandoned by their husbands, denied property, and ostracized by their community [20].

#### The right to HIV counseling

Frequently women reported not receiving pre and post-test counseling as required by the international agreements and the National AIDS Control Organization guidelines [9,10]. This happened more frequently if they attended private clinics and hospitals, as compared with government ICTC or NGOs working in the field of HIV. Eleven of the 14 participants who were tested for HIV in private facilities reported not being counseled before or after testing. A participant described the HIV testing process at an outside laboratory:

Interviewer: Did you know what HIV was?

Participant: No, I had no knowledge about that then. They just said go to the testing center and get all the tests.

Interviewer: So what happened when you went to the testing center [laboratory]?

Participant: They took the blood and they told me to come the next day for the result.

Interviewer: They didn’t explain what the tests were for?

Participant: No. Even when the results came, it was not known to me, until I went to the Doctor.

The lack of pre and post-test counseling was not confined to laboratories. A participant attending a private hospital had a similar experience:

Interviewer: Did you know they were testing you for HIV?

Participant: No. They just said, “Go to that waiting room,” and we went there. There was a sister [nurse] and she took the case paper. We were called and she took the blood.

Interviewer: Did you get the results that day?

Participant: No. We came back the next day.

Interviewer: What happened when they gave you the results?

Participant: They said I should talk to a doctor. He said, “You have the HIV. We cannot touch you here.”

Interviewer: He said it in that way? Did he explain what he meant?

Participant: No. He just gave me a piece of paper and said to go to the government hospital.

In contrast, a woman attending a government ICTC for HIV testing described a very different experience:

Interviewer: She [HIV counselor in a government ICTC] said you should get an HIV test. Then did they tell you why you needed this test?

Participant: So we could get proper treatment for the baby in the future and to prevent the transmission from mother to baby…

Interviewer: How did she tell you that you were [HIV] positive?

Participant: She told that HIV was in the blood.

Interviewer: When the madam [counselor] told you have an HIV infection, were you alone with her?

Participant: I was alone. She asked me whether she should talk to me alone or along with my husband.

Interviewer: Was the test result explained to you in simple language?

Participant: She explained everything so well that I could not think of any questions.

Women in this sample reported starkly different experiences depending on whether they were tested for HIV in private as compared with public facilities. Except for one participant who reported having received HIV counseling at a private nongovernmental organization, none of the other women who had an HIV test outside of a government facility reported being counseled either before or after being tested.

#### The right to be connected to care

All of the women in our sample were eventually linked to PMTCT services and HIV treatment according to standards described in the World Health Organization’s Statement on HIV testing and counseling and the Government of India NACO Guidelines [7,21]. Unfortunately, this usually only happened after accessing antenatal care or delivery services at a public hospital. Connection to care is a critical human rights issue explicitly addressed in the World Health Organization Statement on HIV testing and counseling since women who are not linked to healthcare services are often lost to follow-up with a consequent higher risk for transmitting HIV to their infant [7]. Additionally, should the infant contract HIV it is unlikely to receive appropriate and timely care without those linkages [22]. Of the 14 participants in this sample, eight received single dose Nevirapine for mother and child, and the remaining six women were put on a first line antiretroviral therapy (ART) regimen on their HIV diagnosis since they met the criteria of having a CD4 count of <350 cells/mm3. Three of the participants arrived at a hospital for delivery without previously being tested for HIV. When found HIV positive they received triple ART prophylaxis at the time of delivery. At end of the study, nine of the 14 participants were on a first line ART regimen to manage their HIV infection.

#### The right to non-discrimination

While most participants reported that they did not feel that they had been discriminated against either before or after an HIV test, interviews suggested certain disturbing behavior that could broadly be described as discrimination as defined by international agreements to which the Government of India is a signatory [23]. Private hospitals and nursing homes for instance, almost always referred HIV-positive patients immediately to a public hospital or nongovernmental organization (NGO), even if they could have provided services. One woman talked about how she was told to go to a local NGO for follow-up:

Interviewer: What happened when you got the HIV test report?

Participant: They gave me the report and sent me to a Doctor.

Interviewer: Was he in the same hospital or a different one?

Participant: He was in the same hospital.

Interviewer: What did he tell you? Did he explain what would happen?

Participant: All he said was: “Go to [local HIV NGO]. They will get you pills. You will not get it here”… he told.

Interviewer: That was all? He didn’t tell you about HIV or what would happen?

Participant: No. He just told the sister [nurse]: “Give her a slip [referral information] and phone number.”

It was also apparent from some of the interviews that some women perceived they were treated differently once they had been given an HIV diagnosis. For instance, one participant expressed her anguish after an interview with an ICTC HIV post-test counselor:

Participant: She said… you must know about HIV already. You were given information yesterday. And then she said… that you have it. They ask me whether I had any affair anywhere. Then they ask all kind of information about me and my husband. I felt very bad. It was a blemish on me.

Interviewer: What do you mean by blemish?

Participant: I mean that they don’t talk to you properly. And people scorned at you a lot. I felt very bad that I have landed in such a scenario.

Interviewer: And what were you thinking about at that time?

Participant: I felt a lot of pain. Why is it that everyone looks at me this way? So I want to commit suicide. I thought I should not live anymore.

One woman reported that a physician in a private hospital told her she would have to go to a nearby public hospital for care:

Interviewer: So were you alone when the doctor told you about the HIV?

Participant: No. I was with my mother when I came to the doctor.

Interviewer: So your mother was with you and what did the doctor say?

Participant: She said we will not touch this girl. When she is [HIV] positive… why should we touch her?

Interviewer: They told you like that?

Participant: Ya… They told me like that…then my mother scolded them. “Why madam [she said], you are a doctor and still you say all these things.”

## Discussion

The findings of this research are similar to findings from studies in other countries that documented poor communication between healthcare providers and women receiving HIV testing as part of their antenatal care; and stigma and discrimination if they were found HIV positive [24-28]. Several studies in other parts of India have also suggested that healthcare providers may not frequently be obtaining informed consent, counseling women before or after testing, or keeping test results confidential [29-31]. In this study, informed consent was nonexistent among private practitioners and communication around HIV testing was at best prescriptive, and at worst coercive and threatening. In addition, women reported that their human rights were frequently violated through breaches of confidentiality, with HIV test results frequently being given to family members without consent. Perhaps most disturbing of all, an HIV diagnosis almost always resulted in what appeared to be a denial of services under the guise of referring the patient to a government hospital or ART Center.

From a human rights perspective, only the experiences of two of 14 participants in this study would meet the minimum standards prescribed by the WHO and the Government of India [7,11]. In other words, these were the only participants reporting that they had received all of the 5 c’s: Informed Consent, Confidentiality, Counseling, Correct Result, and Connection to care. Other participants reported a HIV testing process that was seriously flawed either because the minimum standards for testing were not observed, or there were breaches of confidentiality, stigma, or discrimination in the settings they attended for antenatal care. This study found that the human rights of most participants were violated in multiple ways primarily in private hospitals, laboratories and clinics. Often, tests were carried out without explanation or informed consent. HIV counseling was rare or nonexistent, and results were often delivered without any explanation of their significance. Women often learned of their HIV status with other family members within hearing distance, and sometimes even received the diagnosis after others were told. On the whole, participants in our sample appeared much more satisfied with the services offered in public hospitals, particularly in the light of their previous encounters with private for-profit facilities.

The findings of this study are of particular concern in India since 93% of all hospitals, 64% of all hospital beds, 80-85% of doctors, 80% of outpatients and 57% of in-patients are in the private sector [32]. While it is impossible to generalize the results of a qualitative study, the accounts of women from two large Indian states, Karnataka and Maharashtra, should give cause for concern among policymakers and healthcare providers. While violation of human rights is worthy of consideration on its own merits, the import of these issues to HIV prevention efforts should not be underestimated. There is ample evidence that fear of HIV stigma that results from a lack of confidentiality can have a profound impact on the ability and willingness of people to access and utilize HIV prevention services [33]. Although it was clear from most of the interviews that public facilities appear to be providing HIV counseling and testing in antenatal care that meets the WHO criteria, those women tested in government facilities represent only a small fraction of the total pregnancies each year. For PMTCT coverage to reach the Government of India’s vision of eliminating new HIV infections among children, policymakers in India will have to find ways to improve the quality of services offered in the private for-profit sector.

The qualitative methodology utilized in this study has both strengths and weaknesses. While the purposive sampling methods and small sample size make it impossible to generalize the findings, there are compelling reasons for using these methods in this type of research. As Peter Byrne has observed, “stigma is in the eye of the beholder” so it is important for women to describe their own highly contextualized experiences and perceptions about stigma and discrimination during HIV testing in pregnancy [34]. As Miriam has suggested, such research is useful for “understanding how people interpret their experiences, how they construct their worlds, and what meaning they attribute to their experiences” [35].

## Conclusion

The study raises serious concerns about the human rights practices of many private healthcare facilities offering antenatal care in India. Since for-profit companies are the main entry point for HIV testing of pregnant women in India, improving the quality of services that are offered is important both for protecting the rights of women, and the health of their unborn infants. Furthermore, it raises questions whether HIV positive women are protected from stigma and discrimination in order to encourage uptake of these services. Finally, it is incumbent on the Government of India to ensure that all HIV testing, whether in the public or private sector, is anchored in a human rights approach that protects the rights of women and their infants.

## Abbreviations

AIDS: Acquired immunodeficiency syndrome; ART: Antiretroviral therapy; HIV: Human immunodeficiency virus; HTC: HIV testing and counseling; ICTC: Integrated Counseling and Testing Centers; NACO: National AIDS Control Organization; NGO: Nongovernmental Organization; PLWHA: People living with HIV/AIDS; PMTCT: Prevention of mother to child transmission of HIV; UNGASS: United Nations General Assembly Special Session; UNAIDS: United Nations Program on HIV/AIDS; USD: United States dollars; WHO: World Health Organization.

## Competing interests

The authors declare that they have no competing interests.

## Authors’ contributions

Conception and design of the study: PM, SP, CF. Acquisition of data: VK, SK, NV, RS. Analysis and interpretation of data: PM, KK. Drafting the manuscript: PM, KK. Revising it critically for important intellectual content: PM, KK, SP, CF. Final approval of the version to be published: PM, KK, VK, SK, NV, RS, SP, CF.

## Pre-publication history

The pre-publication history for this paper can be accessed here:

http://www.biomedcentral.com/1472-698X/14/7/prepub
